# Enhanced tumour cell nuclear targeting in a tumour progression model

**DOI:** 10.1186/s12885-015-1045-z

**Published:** 2015-02-21

**Authors:** Michael S Nastasie, Helmut Thissen, David A Jans, Kylie M Wagstaff

**Affiliations:** 1 Nuclear Signalling Laboratory, Department Biochemistry and Molecular Biology, Monash University, Clayton, Victoria 3800 Australia; 2 CSIRO Molecular and Health Technologies, Bayview Avenue, Clayton, Victoria 3168 Australia

**Keywords:** Apoptin, tNTS, Histone, Tumour progression model, Isogenic cell line pair, Cancer targeting, Cell penetrating protein

## Abstract

**Background:**

There is an urgent need for new approaches to deliver bioactive molecules to cancer cells efficiently and specifically.

**Methods:**

Here we fuse the cancer cell nuclear targeting module of the Chicken Anaemia Virus Apoptin protein to the core histones H2B and H3 and utilise them in transfection, protein transduction and DNA binding assays.

**Results:**

We found subsequent nuclear accumulation of these proteins to be 2–3 fold higher in tumour compared to normal cells in transfected isogenic human osteosarcoma and breast tumour progression models. This represents the first demonstration of enhanced nuclear targeting by Apoptin in a tumour progression model, and its functionality in a heterologous protein context. Excitingly, we found that the innate transduction ability of histones could be exploited in combination with the Apoptin nuclear targeting module to effect an overall 13-fold higher delivery of protein to osteosarcoma cancer cell nuclei compared to their isogenic normal counterparts.

**Conclusions:**

This is the first report of cancer-cell specificity by a cell penetrating protein, with important implications for the use of protein transduction as a vehicle for gene/drug delivery in the future, and in particular in the development of highly specific and effective anti-cancer agents.

**Electronic supplementary material:**

The online version of this article (doi:10.1186/s12885-015-1045-z) contains supplementary material, which is available to authorized users.

## Background

Despite modest recent decreases in incidence rates due to preventative life style choices, cancer remains one of the most prevalent causes of death in both developed and developing countries [1-3]. The principal difficulty in achieving efficient anti-cancer treatment, whether by chemotherapy, radiotherapy or even excision surgery, has always been in administering the therapy specifically to tumour cells without affecting healthy bystander cells [4-8]. Since cancer cells derive from normal cells and as such share numerous biological/genetic features, targeting one without affecting the other is extremely difficult.

A protein that has attracted interest in this context is viral protein 3 (VP3, also known as Apoptin) from chicken anaemia virus (CAV), which shows enhanced nuclear targeting abilty in tumour compared to normal cells [9-12]. In most cases, Apoptin can also elicit apoptosis in cancer cells [11,13], whereas nuclear translocation of Apoptin does not appear to be sufficient to mediate apoptosis in normal cells [14]. Interestingly, Apoptin nuclear translocation can be triggered in response to DNA damage in normal cells, while blocking these pathways in transformed cells can rescue them from Apoptin-induced cell death [13]. The N-terminal 69 amino acids of Apoptin appear to be responsible for mediating at least some of Apoptin’s cell death pathways [14], whereas the tumour selective nuclear targeting ability of Apoptin has been localised to its C-terminal domain (amino acids 74–121) [15-17], known as the tumour cell enhanced nuclear targeting signal (tNTS). The tNTS contains two NLS regions (a.a. 82–88; 111–121) [14,18,19], as well as an exportin-1 (CRM-1) recognised nuclear export sequence (NES a.a. 97–105) which is inhibited by specific phosphorylation of threonine 108 in cancer cells [15,16,18,20]. Importantly, the Apoptin tNTS region accouns for only ~20% of Apoptin’s proapoptotic ability in cancer cells [14]. The ability of the tNTS to confer cancer cell enhanced nuclear targeting with only minimal toxicity makes it an attractive targeting moiety because of this additional layer of safety by comparison with full length Apoptin. The Aptoptin tNTS is unique in terms of potential as the basis of cancer therapies specifically targeting the nuclei of tumour cells [3,9].

In this study we examine the tumour-selective nuclear targeting ability of the Apoptin tNTS in isogenic tumour and normal cells as well as an isogenic tumour progression cell model. Since the lines derive from the same genetic background, conclusions can be drawn relating directly to tumorigenic status, without confounding differences in genetic or cell type factors. Utilising this powerful system, we show that the tumour-selective nuclear targeting ability of the Apoptin tNTS can function in the heterologous protein context of core histones; core histones are strongly nuclear [21], being responsible for DNA compaction in the nucleus of all eukaryotic cells, meaning that this is, the first demonstration of the ability of the tNTS to function in the context of a highly nuclear protein. In addition, we investigate tNTS activity in the MCF10A breast tumour progression model, demonstrating selectivity for well-progressed tumour phenotypes and highlighting its potential for use in specific anti-cancer treatments in the future. Finally, we show that the tNTS is functional in tumour-selective nuclear targeting in the context of bacterially expressed recombinant histones that retain protein transduction properties, and hence have exciting therapeutic potential. Histones are well-established as being able to undergo protein transduction, and hence possess utility as gene/drug delivery vehicles. The addition of the tNTS to histones thus represents an exciting step forward in the development of tumour-specific anti-cancer therapies [21-25].

## Methods

### Generation of GFP-fusion protein mammalian cell expression plasmids

Gateway cloning technology and the pDest53 vector (Invitrogen) were used to generate mammalian expression vectors encoding green fluorescent protein (GFP) fused to Xenopus histones H2B or H3. The optimised nuclear localisation signal (NLS) from the SV40 large tumour antigen (Op-T-NLS) or the C-terminus of Apoptin (residues 74–121; tNTS) were fused to either histone H2B or histone H3 using primers designed for overlap extension polymerase chain reactions. These were then introduced into either the pDest53 or pTrcHisA-EGFP vector (Invitrogen and Novagen respectively) using Gateway cloning technology as previously described [21]. The fidelity of all constructs was confirmed by DNA sequencing (see Additional 1: Figure S2C). pEGFP (Clontech) and pEPI-tNTS [16] vectors were used to express GFP and GFP-tNTS in mammalian cells respectively.

### Mammalian cell culture and transfection

SAOS-2 cells were cultured in McCoys 5A medium (modified) (Sigma-Aldrich) supplemented with 2.2 g/l sodium bicarbonate, 10% heat inactivated foetal calf serum (FCS) and 1 mM sodium pyruvate. SR5 and SR40 cells were cultured in the above medium with an additional 800 μg/ml Geneticin (GIBCO) as a selective agent. MCF10A, MCF10AT and MCF10CA1h cells were cultured in DMEM/F12 medium with 10 mM HEPES (Sigma), supplemented with 2.2 g/l sodium bicarbonate, 5% horse serum, 100 ng/ml cholera toxin (Sigma), 0.5 μg/ml hydrocortisone, 10 μg/ml bovine insulin and 20 ng/ml human epidermal growth factor (EGF) (GIBCO). All cells were grown at 37°C in 5% CO_2_. Cells were seeded onto glass coverslips (13 mm in diameter or 15 X 15 mm) 24 h prior to transduction experiments or transfection conducted using 4 μg of DNA complexed with 10 μl LipofectAMINE 2000™ (Invitrogen) per well of a 6-well tissue culture plate, according to the manufacturer’s instructions.

### Microscopy and image analysis

Cells were imaged 6–9 hours post transfection by confocal laser scanning microscopy (CLSM; Olympus FV1000) using a 100 X oil immersion objective. The ImageJ v1.43u public domain software was used to analyse digitised images to determine the ratio of nuclear (Fn) to cytoplasmic (Fc) fluorescence ratio (Fn/c) according to the formula: Fn/c = (Fn-Fb)/(Fc-Fb), where Fb is background autofluorescence [21]. An Fn/c value > 1 indicates nuclear accumulation, whereas < 1 indicates cytoplasmic localisation. An Fn/c value of 1 indicates diffuse localisation, with an equivalent amount of protein in both the cytoplasm and the nucleus. To perform a student’s t-test for statistical analysis, Microsoft Excel™ software was used.

### Recombinant protein expression AND purification

The fusion proteins GFP-H3, GFP-H3-tNTS or GFP-H2B-tNTS (expressed from plasmid pTrcHisA-EGFP (Novagen) [26]) were expressed and purified from bacteria as (His)_6_-tagged proteins using nickel affinity chromatography under denaturing conditions (8 M urea) and proteins were eluted in dimerization buffer as previously described [21,23]. Protein purity was c. 70%, based on Coomasie staining of SDS-PAGE (see Additional 1: Figure S2A); although limited degradation products were present, only full-length protein was incorporated into DNA binding histone tetramers (Additional 1: Figure S2B). (His)_6_-tagged GFP protein was made as previously [27].

### DNA electrophoretic mobility shift assay

Recombinant proteins at increasing concentrations were incubated with 300 ng of linearised plasmid DNA (6.7 KB) for 30 minutes at room temperature. Samples were then subjected to electrophoresis in a 0.8% ethidium-free agarose gel at 40 V for 16 h at 4°C in TAE (40 mM Tris, 0.114% glacial acetic acid, 1 mM ethylenediaminetetraacetic acid (EDTA), ph7.5). After staining with ethidium bromide, the gel was visualised under UV illumination.

### Protein transduction

Cells seeded on 13 mm diameter glass coverslips in 24 well plates and 500uL media, were treated with 100uL of 7uM GFP-fusion protein in DMEM for 4 hours at 37°C. The media was removed and the cells washed using phosphate buffered saline (PBS) kept at 37°C to remove free protein. Cells were then treated with DMEM lacking phenol red, containing 1.25ug/mL Hoescht stain for 30 minutes. After thorough washing in a pool of warm PBS and imaging using CLSM as per mammalian cell transfection (above), ImageJ was used to convert the ‘.oif’ files into Tiff format. CellProfiler (r10997) public domain software was used to automatically identify nuclei based on Hoescht staining in the blue channel and measure the relative concentration (as mean fluorescence) of recombinant protein present in those areas using GFP fluorescence indicated in the green channel.

### Additional methods

Please see Additional file 2 for materials and methods related to the Supplementary Figures.

## Results

### The Apoptin tNTS confers tumour cell selectivity to histones H2B and H3

**Figure 1:**
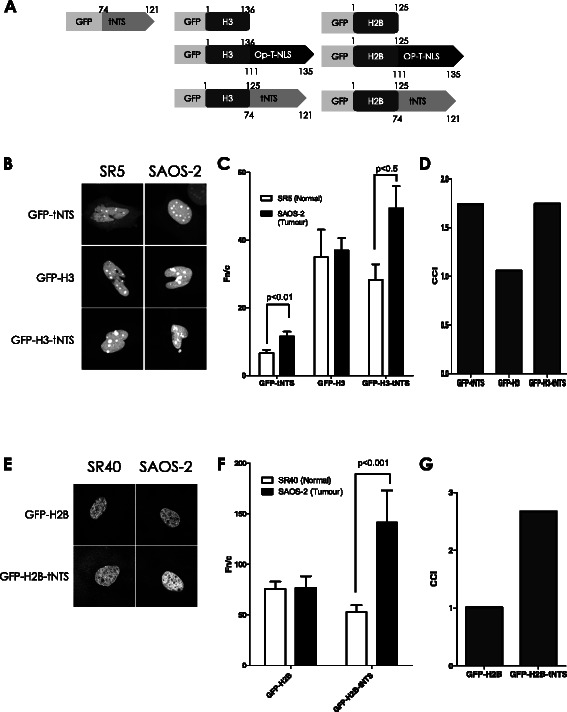
**The tNTS confers tumour cell selectivity to core histone variants. A.** Schematic representation of the engineered histone H2B and H3 proteins utilised in this study. GFP, Green Fluorescent Protein; H2B, histone H2B; H3, histone H3; Op-T-NLS, optimised NLS from the SV40 large tumour antigen (T-ag); tNTS, C-terminal domain of Apoptin (aa 74–121). **B.** SR5 (normal) or SAOS-2 (tumour) cells were transfected to express the indicated fusion proteins followed by CLSM analysis 6 h post-transfection. **C.** Analysis of digitised images such as those shown in **B** was used to determine the relative levels of nuclear accumulation as described in materials and methods. Results represent the mean Fn/c ± SEM (n > 10) and *p* values indicate significant differences as determined by the students t-test. **D.** Cancer comparative indexes (CCI) of the results shown in **C**, determined from a ratio of the Fn/c values. **E.** SR40 (normal) or SAOS-2 (tumour) cells were transfected to express the indicated fusion proteins and imaged by CLSM 6 h post-transfection. **F.** Images such as those in **E** were analysed as per **C** to determine the Fn/c ratio. **G.** CCI’s calculated as per **D** from the results in **F**.

Although the Apoptin tNTS has been shown to be able to confer tumour-cell selective nuclear targeting onto heterologous proteins such as GFP [18,28], it has never been confirmed to confer tumour-cell selectivity to proteins that are intrinsically able to accumulate to high levels in the nucleus. Due to their essential role in DNA compaction and transcriptional regulation, histone proteins contain very strong endogenous NLSs and undergo rapid nuclear import during s-phase such that they are found exclusively in the nucleus and do not normally undergo nuclear export [29]. We generated mammalian and bacterial expression vectors which contain a GFP moiety fused to the N-terminus of either histone H2B or H3 and which also contain either the Apoptin tNTS or an optimised version of the SV40 large tumour antigen NLS (Op-T-NLS) fused to their C-terminus (see Figure 1A for schematic).

For rigorous assessment of tumour cell selective targeting, we utilised isogenic normal/tumour cell line pairs that are derived from a common origin and differ only in their tumorigenic status, including the well-characterised SR5 (SR40)/SAOS-2 osteosarcoma cell pair, which has previously been used to characterise the Apoptin tNTS [16]. SAOS-2 cells contain a nonsense mutation in the retinoblastoma (Rb) gene, with ectopic expression of full length Rb restored in the SR5 and SR40 non-tumorigenic cell lines [30-33], where stable expression of wild type Rb for up to four months is known not to cause any detrimental effects, apart from an increase in cell size and a significant decrease in proliferation [30] consistent with Rb’s cell cycle role [32,33]. We also used a breast tumour progression model, based on the spontaneously immortalized non-tumorigenic MCF10A normal human breast epithelial line [34]. MCF10A cells have been transformed to express the activated c-Ha-ras oncogene to generate the pre-malignant MCF10AT cell line [35,36], which in turn has been used to generate the MCF10CA1h cell line subsequent to selection through xenograft implant in mice [37]. The MCF10AT cell line expresses significantly higher levels of p21 ras protein, as well as the ability to grow in anchorage independent fashion in the absence of growth factors [36]. MCF10AT are pre-malignant, able to generate only small nodules in nude mice, representative of benign simple ducts, with < 25% of xenografts progressing to carcinomas [35,36]. In contrast to the MCF10AT line, MCF10CA1h xenografts rapidly form well-differentiated large carcinomas in 100% of mice with no evident precursor stage [37]. This set of cell lines represents a wide spectrum of neoplastic progression including benign, premalignant and malignant cell lines, and has previously been used to elucidate differences between normal and cancer cell nuclear import [37,38].

SR5 and SAOS-2 cells were transfected to express GFP-H3, GFP-H3-tNTS and GFP-tNTS and examined live using confocal laser scanning microscopy (CLSM; Figure 1B). As has been previously reported, GFP-tNTS alone accumulated in the nucleus of the tumorigenic SAOS-2 cells to a greater extent than in the non-transformed SR5 line, as evident from the increased cytoplasmic fluorescence. This was confirmed by image analysis of digitised images to measure the nuclear to cytoplasmic fluorescence ratio (Fn/c, Methods: Figure 1C; where Fn/c > 1 indicates nuclear accumulation), indicating a significantly higher level (c. 2 fold, p < 0.01) of nuclear accumulation in the tumorigenic SAOS-2 cells, compared to the non-transformed SR5 cells. In contrast, GFP-H3 was almost exclusively nuclear in both lines, with little to no visible cytoplasmic fluorescence evident (Figure 1B). Quantitative analysis confirmed that there was no significant difference in the nuclear accumulation of GFP-H3 between the two cell lines. Excitingly, the addition of the tNTS to GFP-H3 significantly increased the nuclear accumulation of the histone in the tumorigenic SAOS-2 line and not in non-transformed SR5 cells, indicating that the tNTS can confer tumour-cell selective activity even when in the context of a highly nuclear heterologous protein, indicating for the first time that histones can be manipulated to target preferentially to the nuclei of tumour cells. The Cancer Comparative Index (CCI) [39], the ratio of the Fn/c of the protein in tumour compared to normal cells (where a CCI greater than 1 denotes increased nuclear accumulation in tumour cells), was 1.7 for GFP-tNTS (Figure 1D), consistent with previous observations [16,39], whilst GFP-H3 had a CCI of c. 1.0, indicating no cancer specificity. In contrast, GPF-H3-tNTS had a CCI of 1.8, comparable to that for GFP-tNTS, demonstrating clearly that tumour-selective targeting can be transferred to nuclear proteins such as histones.

To confirm that the enhanced tumour-cell selectivity shown here is not a specific property of histone H3, the core histone H2B was analysed in identical fashion (Figure 1E-G). In comparable fashion to the results for H3, GFP-H2B was predominantly nuclear to the same extent in SR40 and SAOS-2 cells (CCI of 1.0), but the presence of the tNTS resulted in significantly higher nuclear accumulation in SAOS-2 transformed cells (CCI of 2.7, p < 0.001). Clearly, the Apoptin tNTS has enhanced cancer cell nuclear targeting ability that is transferrable to proteins such as histones H3 and H2B, that normally have no inherent ability to accumulate to a higher extent in the nuclei of tumour versus normal cells.

### Apoptin tNTS enhanced nuclear targeting in a tumour progression model

**Figure 2:**
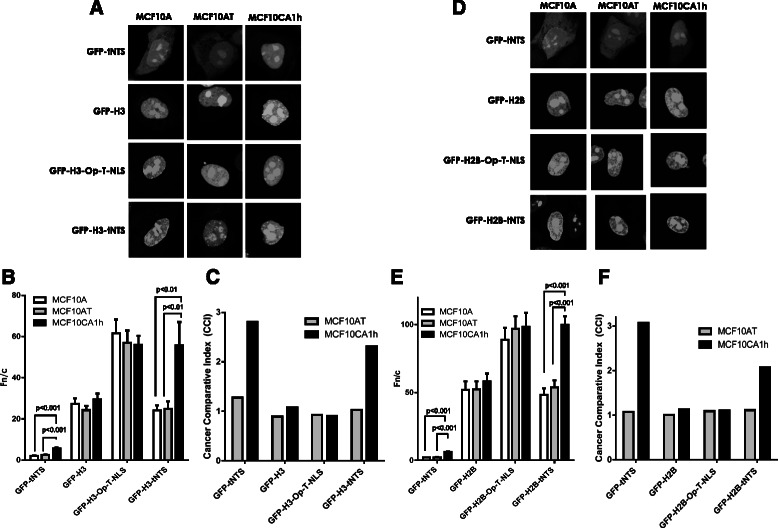
**Tumour cell enhanced nuclear targeting conferred by the Apoptin-tNTS is observed in a breast cancer tumour progression model and can be conferred to histones H3 and H2B. A.** MCF10A (normal), MCF10AT (pre-malignant) or MCF10Ca1h (tumour) cells were transfected for 6 h to express the indicated fusion proteins followed by CLSM analysis. **B.** Digitised images as those shown in **A** were analysed to determine relative levels of nuclear accumulation displayed as mean Fn/c ± SEM (n > 40) where *p* values indicate significant differences determined by the students t-test. **C.** CCIs of the results shown in **B**, determined from a ratio of the Fn/c values. Results are from a single typical experiment from a series of three similar experiments. **D.** MCF10A (normal), MCF10AT (pre-malignant) or MCF10Ca1h (tumour) cells were transfected for 6 h to express the indicated fusion proteins. **E.** Digitised images as those shown in **D** were analysed to determine relative levels of nuclear accumulation displayed as mean Fn/c ± SEM (n > 40) where *p* values indicate significant differences determined by the students t-test. **F.** CCIs of the results shown in **E**, determined from a ratio of the Fn/c values. Results are from a single typical experiment from a series of two similar experiments.

We next tested tNTS dependent targeting in the MCF10A breast tumour progression model. CLSM analysis of transfected cells expressing the GFP-tNTS construct alone revealed a significant (p < 0.001) increase (almost 2-fold) in nuclear accumulation for GFP-tNTS in MCF10CA1h tumorigenic cells compared to both normal MCF10A and pre-malignant MCF10AT cells (Figure 2A-C). The almost 3 CCI indicated high cancer cell specificity, the first demonstration of tumour cell specific targeting by the Apoptin tNTS in a human breast cancer progression model. That the Apoptin tNTS displays a strong preference for more advanced tumour phenotypes, highlights its potential effectiveness as a tumour targeting moiety for cancer-therapeutics.

To confirm that the tumour selective activity of Apoptin can be conferred to histone proteins in this breast tumour model system, the engineered histone H3 constructs were expressed in the MCF10A isogenic cell series. As observed for the SR5/SAOS-2 cell line pair, GFP-H3 did not show a significant difference in nuclear accumulation across the three cell lines. In contrast, GFP-H3-tNTS showed significantly (p < 0.01) higher nuclear accumulation in MCF10CA1h cells compared to either the MCF10A normal or the MCF10AT pre-malignant cells (Figure 2B). Further, the CCI of GFP-H3-tNTS suggests that the protein accumulates to a >2 fold higher extent in the nuclei of the tumorigenic MCF10CA1h cells compared to the MCF10A, non-transformed cells (Figure 2C). Excitingly, this demonstrates for the first time that the tumour cell nuclear targeting ability of the tNTS can be transferred to heterologous proteins in a model which closely resembles human proliferative breast disease with increasing targeting preference in the more advanced stages of breast tumour progression. To demonstrate that this activity is exclusively dependent on the tNTS sequence and not merely the result of the addition of an exogenous NLS to the histone protein, we replaced the Apoptin tNTS with the optimised version of the SV40 large-tumour antigen (Op-T-NLS), which has previously been shown to increase the nuclear accumulation of other core histone proteins [21]. Although, the nuclear accumulation of GFP-H3-Op-T-NLS was significantly elevated compared to GFP-H3 alone, the increase was comparable across the MCF10A, MCF10AT and MCF10CA1h cell lines, with no significant difference in Fn/c observed (Figure 2B, C), confirming that the tumour-cell selective activity of these novel histones is not a general feature of NLSs but a unique property of the Apoptin tNTS.

### Tumour-cell selective nuclear targeting can also be conferred to histone H2B in the tumour progression model

To confirm that the results observed in the breast tumour progression model were not specific to histone H3, histone H2B was also examined in the same way. Whereas GFP-H2B and GFP-H2B-Op-T-NLS showed no significant difference in their nuclear accumulation between the MCF10A, MCF10AT and MCF10CA1h cells (Figure 2D-F), the nuclear accumulation of GFP-H2B-tNTS was significantly (p < 0.001) higher in the tumorigenic MCF10CA1h cells compared to the other cell types, with a CCI of >2 (Figure 2C). The level of tumour cell selectivity observed for GFP-H2B-tNTS is nearly identical to that observed for GFP-H3-tNTS in this cell system, implying that tumour cell selective activity is derived specifically from the Apoptin tNTS. It thus seems likely that robust tumour-cell specificity can be conferred by the tNTS to other heterologous proteins.

### Recombinant histone proteins retain DNA binding ability when fused to the tNTS

**Figure 3:**
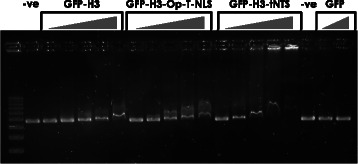
**Engineered histone H3 proteins bind to DNA.** The indicated recombinant proteins were incubated at increasing concentrations (left to right; 0.1, 0.25, 0.5, 0.75 and 1 μM for the histone proteins and 0.5 and 1 μM for GFP) with 300 ng of linearised plasmid DNA (6.7 KB) for 30 min at room temperature prior to electrophoresis (40 V) at 4°C for 16 h against DNA alone (−). GFP alone was also incubated with DNA in the same way. Results are from a single typical experiment from a series of two similar experiments.

The ability of histones to bind DNA is critical to their use as DNA delivery vectors for future gene therapy applications [21]. To confirm that the DNA binding capacity of our novel tumour selective fusion proteins is not impaired by the addition of the tNTS, recombinant GFP-H3, GFP-H3-Op-T-NLS and GFP-H3-tNTS fusion proteins were examined in a DNA electrophoretic mobility shift assay. Linearised plasmid DNA was incubated with increasing concentrations of the recombinant proteins and subjected to agarose gel electrophoresis followed by ethidium bromide staining and UV illumination to visualise the DNA bands (Figure 3). DNA binding in this assay is evidenced by a retardation of the movement of the DNA through the agarose gel. GFP-H3 and GFP-H3-Op-T-NLS were able to retard the movement of the linearised DNA to a similar extent, indicative of comparable DNA binding ability. Interestingly, GFP-H3-tNTS DNA binding ability appeared to alter bands whilst at lower concentrations of protein, indicating a higher binding to DNA. This is consistent with wild type histone H3 having the highest DNA binding ability of the four core histones, whereby histone H3 binds to the DNA so strongly that it is unable to enter the gel and remains in the well (Additional 1: Figure S1).

### Histones containing the tNTS can enter into intact cells via protein transduction and exhibit tumour specific nuclear targeting ability

**Figure 4:**
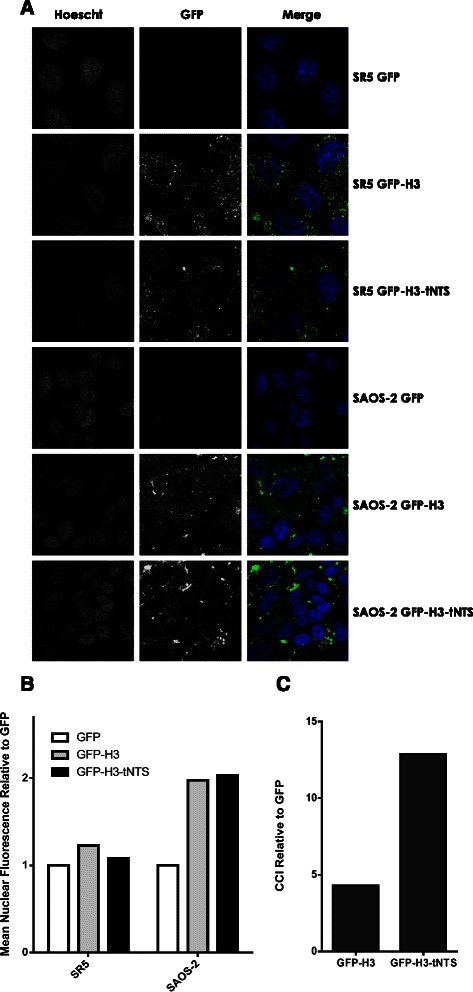
**Innate tumour cell selectivity is exhibited by the recombinant histone H3 protein and is further enhanced by the Apoptin-tNTS. A.** SR5 (normal) or SAOS-2 (tumour) cells were incubated for 4 hours in media containing 1.17 μM indicated recombinant protein. Cells were then washed with warm 37°C PBS and treated with DMEM lacking phenol red, containing 1.25 μg/mL Hoescht stain for 30 min. Cells were thoroughly washed in warm PBS and imaged using CLSM as per mammalian cell transfection (above). The blue channel shows Hoescht stained nuclei and the green channel shows the uptaken GFP-tagged recombinant protein. Blue (Hoescht) and Green (GFP) channels are presented separately in grayscale and merged in colour for clarity. **B.** Analysis of digitised images such as those in **A** was used to determine the relative mean fluorescence in the nucleus of cells as described in Materials and Methods. The mean fluorescence value was then normalised to the GFP value representing background fluorescence in each cell line. **C.** The data in **B** was used to calculate CCI values for GFP-H3 and GFP-H3tNTS recombinant proteins, relative to GFP alone (as disregarded background). Results are from a single typical experiment from a series of two similar experiments.

Histones are members of the cell penetrating family of proteins possessing innate protein transduction ability, enabling them to pass through the plasma membrane of cells in an energy and receptor-independent mechanism, a further property of great utility in gene therapy applications [21,23,40,41]. Based on the high CCI index observed in transfected cells, the GFP-H3 and GFP-H3-tNTS based constructs were selected to test whether the tNTS fusion proteins generated here have potential for tumour selective delivery in a protein transduction assay. SAOS-2 or SR5 cells were incubated with purified recombinant GFP, GFP-H3 or GFP-H3-tNTS recombinant proteins. Cells were then washed extensively to remove extracellular protein, stained with Hoechst and imaged live via CLSM (Figure 4A). As GFP itself does not possess any natural protein transduction potential the level of fluorescence in the nucleus of these samples did not differ significantly from the PBS alone control (not shown), as expected. Strikingly, the level of fluorescence of GFP-H3 quantitated in the SAOS-2 transformed cell line nuclei was increased by c. 350% compared that observed in the non-transformed counterpart (after taking into account background) (Figure 4B) which represents a CCI of > 4 (Figure 4C), indicating tumour specific nuclear accumulation in the absence of the tNTS, most likely attributable to differences in the protein transduction capacity of GFP-H3 in the two lines, with much more total protein taken up by the tumour cells in general thereby leading to a higher level of nuclear fluorescence (Figure 4A). This is the first report of cell specificity for histone-mediated protein transduction, which had previously been thought to be similar in all cells [21,41]. Excitingly, this is also the first report of innate tumour cell selectivity for a protein transduction domain containing protein. This result was confirmed by subcellular fractionation experiments (Additional 1: Figure S3), whereby GFP-H3 and GFP-H3tNTS could not be detected in the SR5 normal cells under the conditions used, but were readily observed in the tumour cells.

GFP-H3-tNTS showed an even more dramatic difference in the level of nuclear fluorescence in the tumour cells compared to normal cells (Figure 4A), with a CCI of 13 (Figure 4C). Similar results were observed for GFP-H2B-tNTS (CCI of 11, not shown), confirming that this activity is not specific to histone H3. It is interesting to note that the additional tumour selective ability conferred by the tNTS did not appear to be due to an increase in protein concentration in the nucleus of the transformed SAOS-2 cells, but rather, was the result of a decrease in protein concentration in the nucleus of the normal SR5 cells (Figure 4B). These results confirm that the nuclear export activity of the tNTS on highly nuclear proteins such as histones in normal cells is retained in the purified recombinant fusion proteins and furthermore this affect is enhanced synergistically when using proteins that already harbour innate tumour cell selectivity as was observed here for the first time for histones.

## Discussion

Current cancer treatments are often associated with debilitating side-effects on the surrounding healthy tissue and ultimately the patient due to the lack of specificity of anti-cancer chemotherapy, radiotherapy or amputation/excision surgery, which take advantage of specific attributes endemic to tumours such as an increased level of cellular proliferation, but which are often also present in normal tissues, such as the rapidly proliferating cells of the bone marrow. The Apoptin protein from chicken anaemia virus however shows selectivity in tumour compared to normal cells, with increased nuclear accumulation in tumour cells. Here we investigate a novel use of the Apoptin protein, by isolating its tumour cell selectivity and utilising it to generate tumour-cell selective proteins. We used a human breast cancer tumour progression model, to examine the nuclear localisation of the Apoptin tNTS for the first time, showing that the tNTS is significantly more nuclear in the tumorigenic MCF10CA1h cell line compared to the normal MCF10A or the MCF10AT pre-malignant isogenic counterparts. This underlines the fact that Apoptin’s nuclear localization specificity is imparted by the tNTS and can be conferred on heterologous proteins which are normally exclusively nuclear. Presumably this specificity is due to increased export of the Apoptin tNTS in normal cells [16].

We have shown here using two different normal/tumour isogenic cell type models that the Apoptin tNTS was able to increase the nuclear accumulation of histones H3 and H2B specifically in cancer cells but not in normal isogenic counterparts, with a preference for advanced tumour phenotypes similar to the Apoptin tNTS alone. Whilst the tNTS has previously been fused to GFP and maltose-binding-protein (MBP) for tracking purposes, resulting in their cancer cell specific localisation [15,16,42], it has not previously been fused to a highly nuclear protein such as a histone. Thus, we show here for the first time that the tNTS is sufficient to impart cancer cell nuclear specificity upon histone proteins and we predict that this will be consistent for other nuclear proteins, which could have a great impact on the development of future specific nuclear delivery of cancer therapeutics.

In this study we were also able to demonstrate that the addition of the tNTS moiety enhances the DNA binding ability of the recombinant GFP-tagged histone protein, indicating that the tNTS moiety itself is capable of mediating some DNA binding. The fact that the Op-T-NLS, containing several positive residues, does not enhance DNA binding here indicates that the contribution to DNA binding shown by the tNTS is specific. This is consistent with previous literature which showed that Apoptin is able to bind DNA [43] and the effect here may be assumed to be a summative one. The implications of this may be far reaching, as previously the focus when using the tNTS was on its cancer specific nuclear delivery capabilities, we show here for the first time that it is likely to provide dual benefit when incorporated into vectors being engineered for gene therapy applications. Similarly, although we have not observed any apoptotic effects in the present study, potential Apoptin-induced tumour-specific apoptosis that might occur in therapeutic applications would have a comparable additive benefit in terms of tumour cell killing.

Perhaps the most interesting finding from this study was that histone H3 exhibits innate cancer specific transduction potential. This is the first report of cancer specific transduction capacity for histones, and for innate tumour selectivity in a protein transduction domain in general, which further highlights their potential as gene delivery vectors, especially with respect to cancer therapeutics. When recombinant fusion proteins were delivered to cells via protein transduction, the addition of the tNTS to histone H3 (or histone H2B, not shown) resulted in a synergistic effect that allowed for 13 fold higher nuclear accumulation of the protein in the transformed SAOS-2 cells compared to the normal SR5 isogenic counterparts. The affect of the tNTS was due to a decrease in protein concentration in the nucleus of the normal SR5 cells rather than an increase in the SAOS-2 transformed cell line. This is consistent with the tNTS being actively exported from the nucleus by an exportin-1 dependant mechanism in normal cells. In transformed cell types, however, nuclear export mediated by the tNTS is inhibited through phosphorylation of Threonine108 [15,16]. The results here suggest that the tNTS is functional even when included within a fusion protein delivery system.

There are many physiological examples in which the extent of nuclear accumulation rather than the simple presence or absence of a protein in the nucleus is a key driver, such as in developmental processes or signal transduction and so these novel vectors may prove quite useful even before complete specificity is achieved. Examples include human sex determination and nuclear concentration of the SRY/SOX9 transcription factors (where c. 2-fold differences in Fn/c represent the basis of male/female sex determination) [44-47] and in neuronal cell function with respect to the NFkB p65 transcription factor (see [48]). The nuclear levels of specific viral proteins have been shown to be critical during viral infection for a range of viral pathogens, including CMV [49], porcine reproductive and respiratory syndrome virus [50], respiratory syncytial virus [51] and dengue [52]. Importantly, threshold levels of nuclear accumulation are critical for effective cancer gene/drug treatment strategies for numerous systems (see [53]), such as the first-line anti-cancer drug doxorubicin [54] meaning that a >10 fold increase in nuclear accumulation exhibited by histones engineered in this study may prove very valuable factors in the future for anti-cancer therapeutics. Using recombinant histone vectors from this study in preliminary gene delivery experiments we have already achieved transfection in transformed cell lines (not shown), further highlighting their potential for use in future cancer targeted gene delivery strategies in addition to their potential for directed transport of drug/peptide cargo to cancer cell nuclei.

## Conclusions

In conclusion, we used here for the first time a human breast tumour progression model to demonstrate that the nuclear accumulation of the Apoptin tNTS is significantly higher in transformed cells compared to their normal isogenic counterparts and that this activity can be conferred to histones H3 and H2B which we have also shown for the first time to possess innate cancer targeting abilities. Whilst these histones may be further developed to increase their specificity for tumours this study highlights the power of the Apoptin tNTS and its utility as a module for the development of a future class of highly specific anti-cancer agents.

## Additional files

Additional file 1: Figure S1. Wild type xenopus histone proteins bind have distinct DNA binding abilties. A DNA gel mobility shift assay was performed using the indicated proteins at increasing concentrations (0, 0.5, 0.75, 1 μM) and 300 ng of linearised plasmid DNA (pUC18) as per the legend to Figure 3. GFP was used only at 1 μM. Results are from a single typical experiment from a series of two similar experiments. **Figure S2.** Validation of recombinant proteins generated in this study. A. 4 and 8 μg of the indicated recombinant proteins was subjected to SDS-PAGE followed by Coomassie staining and imaging on a UV-transilluminating platform as described in materials and methods. M: Page Ruler molecular weight marker B. Histone tetramers containing the indicated recombinant H3 proteins and equimolar wild type Xenopus Histone H4 [2], were separated using SDS-PAGE, stained and visualized as in A. C. Sequence alignments of GFP-H3 (i) and GFP-H3-tTNS (ii) expression plasmids compared to pre-engineered recombinant protein templates as described in materials and methods. **Figure S3.** Nuclear/Cytoplasmic fractionation of SR5 and SAOS-2 cells transduced with recombinant histones demonstrates tumour-cell specificity. SR5 and SAOS-2 cells transduced with the indicated recombinant proteins (as per Figure 4) were subjected to nuclear/cytoplasmic fractionation as described in the Additional file 2: Supplementary materials and methods. Samples were subjected to SDS-PAGE and Western analysis using anti-GFP and anti-actin primary antibodies and fluorescent secondary antibodies.

Additional file 2: **Supplementary Materials and Methods.**

## Abbreviations

CAV: Chicken anaemia virus
CCI: Cancer comparative index
CLSM: Confocal laser scanning microscopy
CRM-1: Lexportin-1
EGF: Epidermal growth factor
FCS: Foetal calf serum
Fn/c: Nuclear to cytoplasmic fluorescence ratio
GFP: Green fluorescent protein
MBP: Maltose-binding protein
NES: Nuclear export sequence
NLS: Nuclear localisation signal
Op-T-NLS: Optimised version of the SV40 large tumour antigen NLS
PBS: Phosphate buffered saline
Rb: Retinoblastoma
T-ag: SV40 large tumour antigen
tNTS: tumour cell enhanced nuclear targeting signal
