# Supplementary material for: Enhanced tumour cell nuclear targeting in a tumour progression model
Source: BMC Cancer. 2015 Feb 21;15:76. doi: 10.1186/s12885-015-1045-z (PMC4342815; doi:10.1186/s12885-015-1045-z)
Supplement: Supplementary file 1 — Wild type xenopus histone proteins bind have distinct DNA binding abilties. A DNA gel mobility shift assay was performed using the indicated proteins at increasing concentrations (0, 0.5, 0.75, 1 μM) and 300 ng of linearised plasmid DNA (pUC18) as per the legend to Figure 3. GFP was used only at 1 μM. Results are from a single typical experiment from a series of two similar experiments. Figure S2. Validation of recombinant proteins generated in this study. A. 4 and 8 μg of the indicated recombinant proteins was subjected to SDS-PAGE followed by Coomassie staining and imaging on a UV-transilluminating platform as described in materials and methods. M: Page Ruler molecular weight marker B. Histone tetramers containing the indicated recombinant H3 proteins and equimolar wild type Xenopus Histone H4 [2], were separated using SDS-PAGE, stained and visualized as in A. C. Sequence alignments of GFP-H3 (i) and GFP-H3-tTNS (ii) expression plasmids compared to pre-engineered recombinant protein templates as described in materials and methods. Figure S3. Nuclear/Cytoplasmic fractionation of SR5 and SAOS-2 cells transduced with recombinant histones demonstrates tumour-cell specificity. SR5 and SAOS-2 cells transduced with the indicated recombinant proteins (as per Figure 4) were subjected to nuclear/cytoplasmic fractionation as described in the Additional file 2: Supplementary materials and methods. Samples were subjected to SDS-PAGE and Western analysis using anti-GFP and anti-actin primary antibodies and fluorescent secondary antibodies. [file 12885_2015_1045_MOESM1_ESM.pptx]

## Slide 1
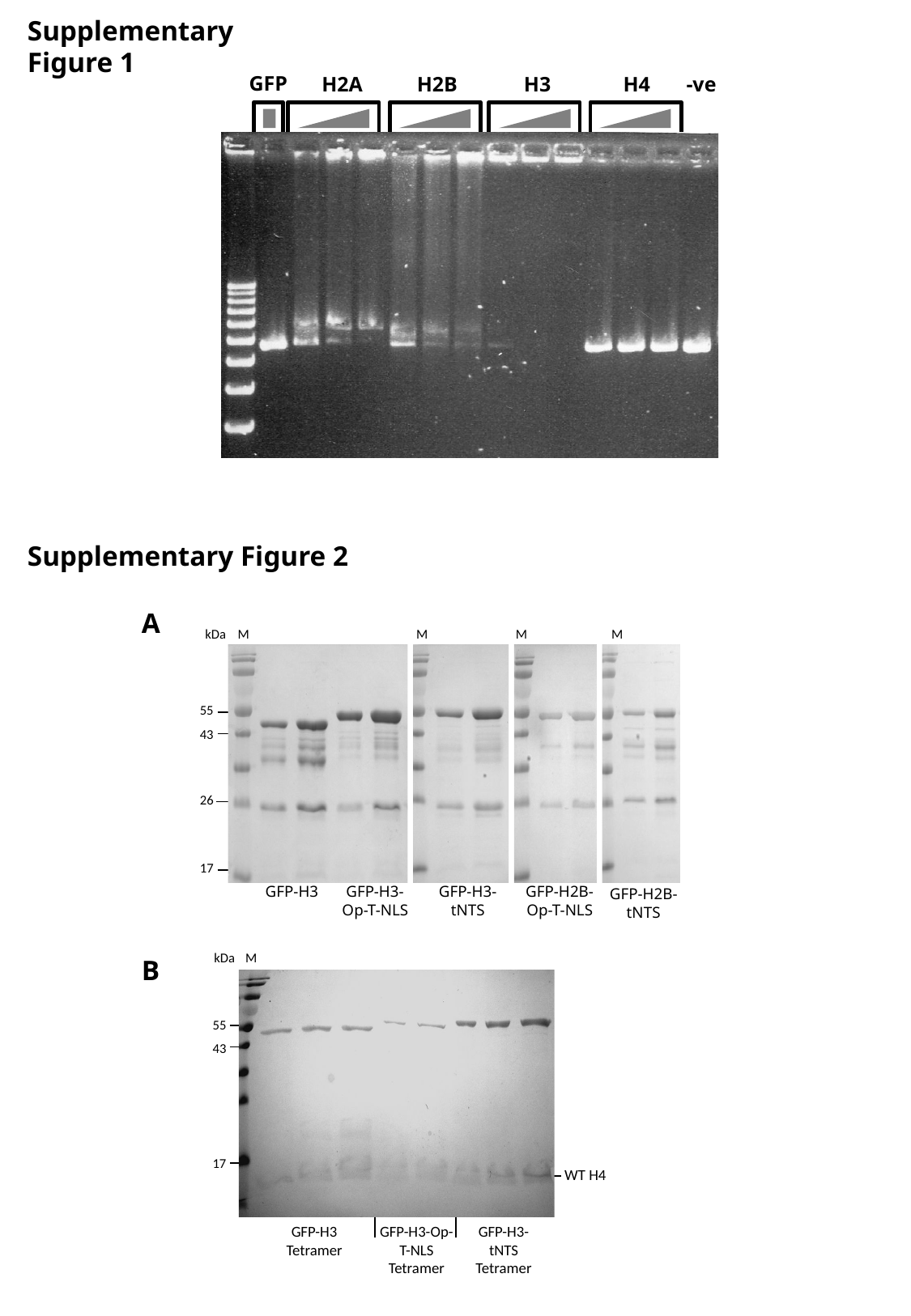

Supplementary Figure 1
GFP
H2A
H2B
H3
H4
-ve
Supplementary Figure 2
A
kDa
M
M
M
M
55
43
26
17
GFP-H3
GFP-H3-Op-T-NLS
GFP-H3-
tNTS
GFP-H2B-Op-T-NLS
GFP-H2B-
tNTS
kDa
M
B
55
43
17
WT H4
GFP-H3
Tetramer
GFP-H3-Op-T-NLS
Tetramer
GFP-H3-tNTS
Tetramer

## Slide 2
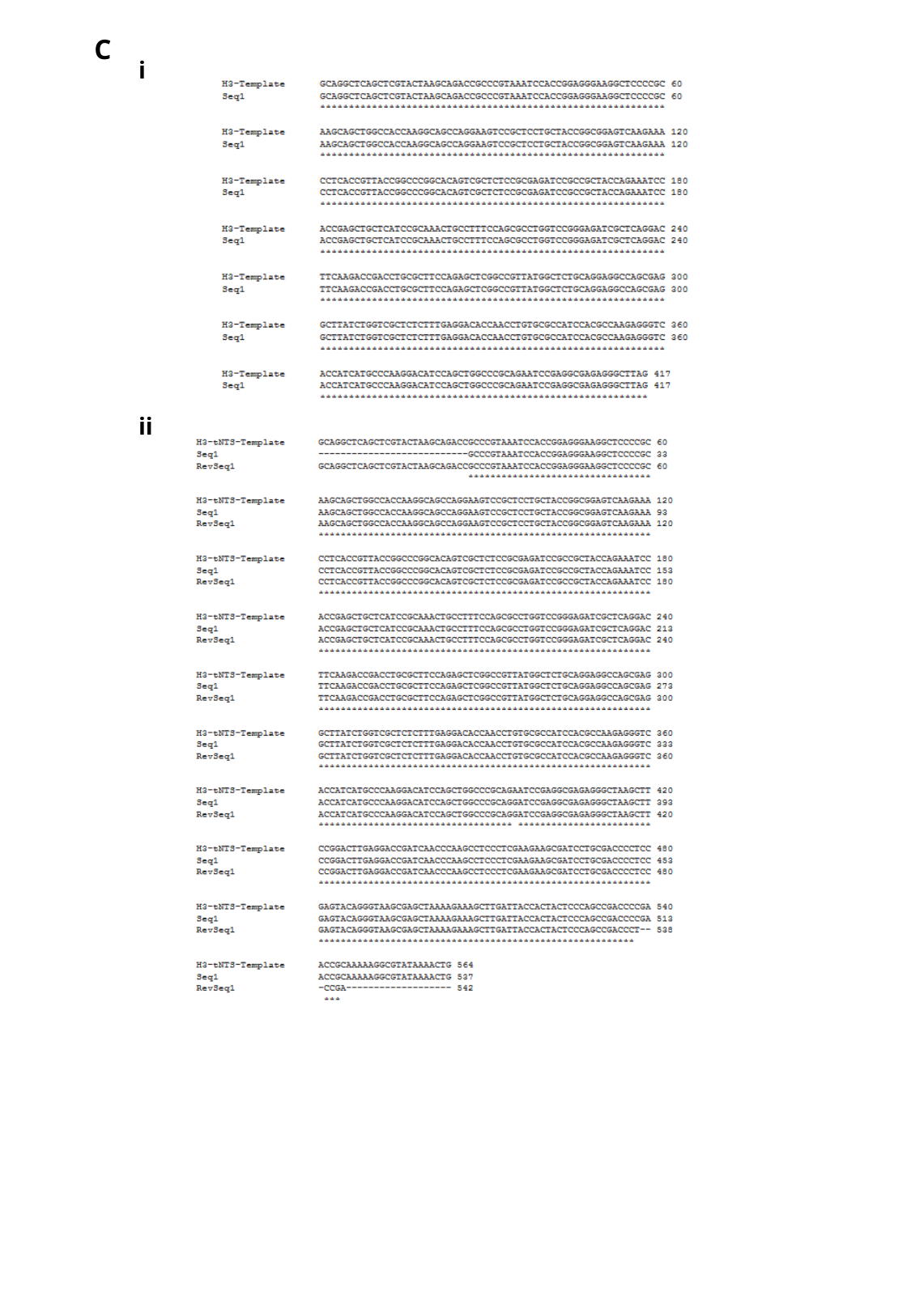

C
i
ii

## Slide 3
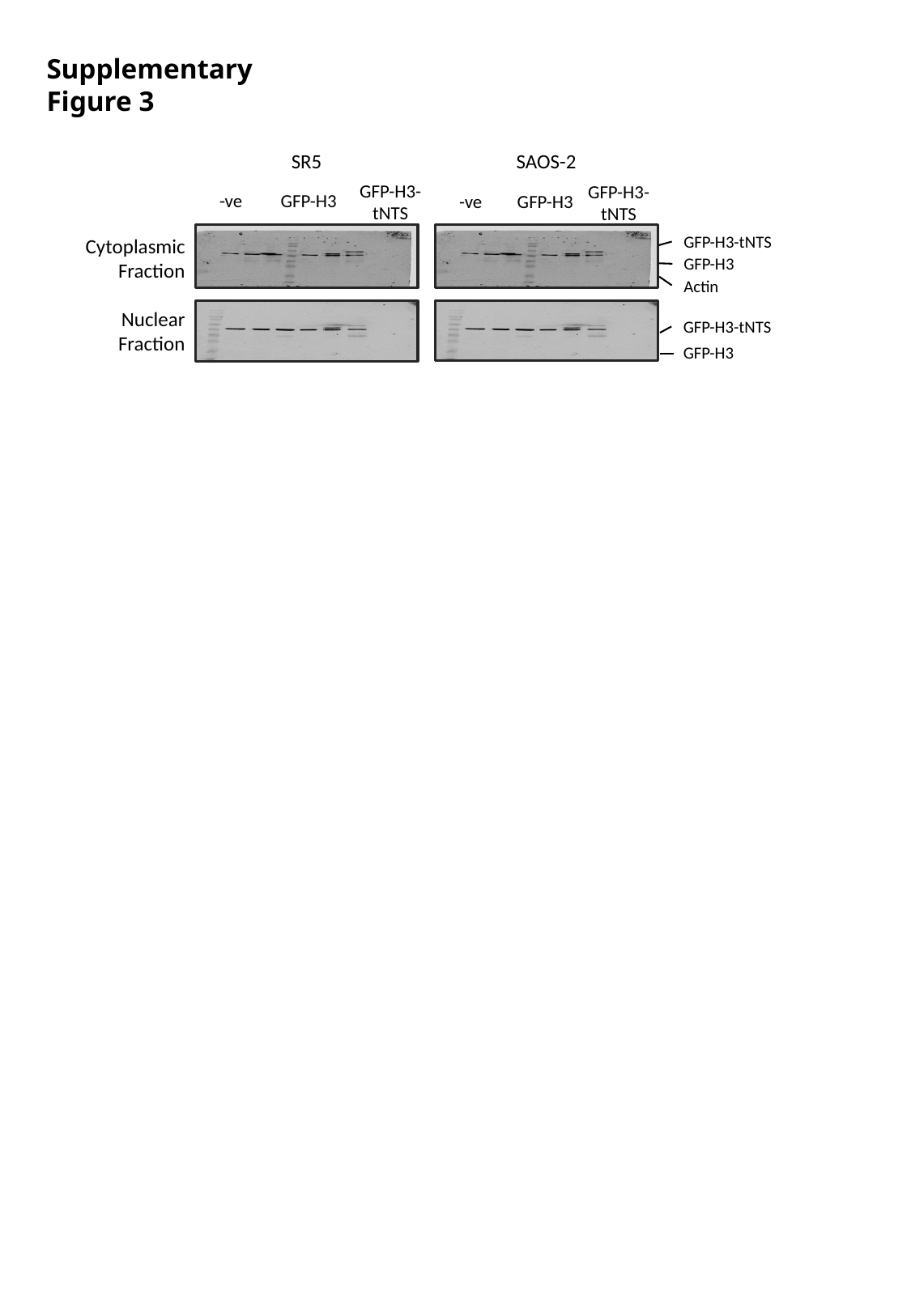

Supplementary Figure 3
SR5
SAOS-2
GFP-H3-tNTS
GFP-H3-tNTS
-ve
GFP-H3
-ve
GFP-H3
GFP-H3-tNTS
Cytoplasmic Fraction
GFP-H3
Actin
Nuclear Fraction
GFP-H3-tNTS
GFP-H3
