# Supplementary material for: Enhanced tumour cell nuclear targeting in a tumour progression model
Source: BMC Cancer. 2015 Feb 21;15:76. doi: 10.1186/s12885-015-1045-z (PMC4342815; doi:10.1186/s12885-015-1045-z)
Supplement: Supplementary file 2 — Supplementary Materials and Methods. [file 12885_2015_1045_MOESM2_ESM.docx]

**Supplementary Materials and Methods:**

*DNA sequencing:*

Sequencing was conducted using BigDye Terminator chemistry according to the manufacturer’s instructions (Applied Biosystems). The sequencing reactions were precipitated by the addition of 3.6 μL of 3 M NaOAc, 60.4 μL 100% Ethanol (EtOH) in 100 μL final volume and incubated for 10 min at room temperature (RT). Precipitated DNA was pelleted at 13000 rpm 25 min at RT, washed once with cold 70% EtOH and left to dry at RT. Samples were submitted for automated sequencing (Micromon facility, Monash University), and the data analysed using ApE© (v2.0.36, M.W. Davis, freeware). Multiple sequencing alignments were conducted using the ClustalW2 web service (EMBL-EBI).

*SDS-PAGE and Coomassie staining*.

Purified proteins were assessed by sodium dodecyl sulfate (SDS)-polyacrylamide gel electrophoresis (SDS PAGE), using a 12% gel. 5 μl PageRuler (Thermo Scientific) was routinely used as a molecular weight marker. Following electrophoresis, the gels were incubated in Coomassie Blue staining solution (0.25% (w/v) Coomassie Brilliant Blue R-250, 45% (v/v) methanol, 7% (v/v) glacial acetic acid) for 1 h at RT and destained for 6-16 h (45% (v/v) methanol, 7% (v/v) glacial acetic acid). Gels were visualised on a UV-transilluminating platform and digitally imaged. The ImageJ v1.43u public domain software was used to conduct densitometric analysis on the digitized images. Total protein concentration was determined using the Bio-rad Protein Assay (Bio-Rad) according to the manufacturer’s instructions.

*Isolation of Cytoplasmic and Nuclear Fractions*

Nuclear fractionation was conducted as previously [1]. Briefly, SR5 or SAOS-2 cells were harvested using 0.02% ethylenediaminetetraacaetic acid (EDTA) in PBS, then washed twice with PBS. Intact nuclei were extracted by suspending the cells in nuclei buffer (NB) (320 mM sucrose, 110 mM KCl, 5 mM NaHCO3, 5mM MgCl2, 1 mM ethylene glycol tetraacaetic acid, 0.1 mM CaCl2, 1 mM dithiothreitol, 20 mM HEPES pH 7.4) supplemented with 0.5% Triton X-100 and incubated on ice for 5 min. Nuclei were pelleted at 2000 x *g* for 5 min at 4°C and the supernatant collected as the cytoplasmic fraction. The pelleted nuclei were washed twice using NB, suspended in 1% CHAPS solution and incubated on ice for 10 min. Nuclear debris was pelleted using high speed centrifugation and the resulting supernatant was used as the nuclear fraction.

*Western blotting*

20 μg of protein from the cytoplasmic fraction and all of the nuclear fraction from each sample was subjected to SDS-PAGE (12% gel) and transferred to nitrocellulose membrane at 300 mA for 3 h in transfer buffer (25mM Tris-base, 192mM glycine, 5% (v/v) isopropanol). The membrane was rinsed in PBS and blocked in PBS/5% skim milk for 1 h at RT, then incubated with primary antibody (anti-H3 diluted 1:3000 (Abcam), anti-GFP diluted 1:3000 (Roche) or anti-actin diluted 1:4000 (Abcam)) in PBST (PBS/0.05% Tween-20) with 2.5% skim milk, for 16 h at 4°C. Membranes were then washed three times with PBST and incubated with secondary antibody (IRDye anti-mouse immunoglobulin (Ig) (800CW) and IRDye anti-rabbit Ig (680RD); LI-COR), diluted 1:4000 in PBST/2.5% skim milk. Following three 15 min washes with PBST the blot was imaged using an Odyssey Infrared Imaging System at 700 nm and 800 nm (LI-COR). Membranes were stripped where necessary using stripping buffer (0.188% glycine, 1% SDS, pH 2) at 65ºC.

1. Glover DJ, Leyton DL, Moseley GW, Jans DA: **The efficiency of nuclear plasmid DNA delivery is a critical determinant of transgene expression at the single cell level.** *J Gene Med* 2010, **12:**77-85.

2. Wagstaff KM, Fan JY, De Jesus MA, Tremethick DJ, Jans DA: **Efficient gene delivery using reconstituted chromatin enhanced for nuclear targeting.** *FASEB J* 2008, **22:**2232-2242.
